# Influence of Heat Treatment on Microstructure and Mechanical Properties of AZ61 Magnesium Alloy Prepared by Selective Laser Melting (SLM)

**DOI:** 10.3390/ma15207067

**Published:** 2022-10-11

**Authors:** Shuai Liu, Hanjie Guo

**Affiliations:** 1 School of Artificial Intelligence, Beijing Technology and Business University, Beijing 100048, China; 2 School of Metallurgical and Ecological Engineering, University of Science and Technology Beijing, Beijing 100083, China; 3 Beijing Key Laboratory of Special Melting and Preparation of High-End Metal Materials, Beijing 100083, China

**Keywords:** selective laser melting, magnesium alloy, heat treatment, microstructure, mechanical properties

## Abstract

From previous studies, it is known that the dissolution of β-Mg_17_Al_12_ at high temperature and the increase of densities at high pressure after hot isostatic pressing (HIP) are the two main reasons for significant improvement in the ductility of AZ61 magnesium alloy prepared by SLM. However, the mechanism of dissolution of β-Mg_17_Al_12_ in SLMed AZ61 magnesium alloy at high temperature is not clear. To illustrate the mechanism of the effect of β-Mg_17_Al_12_ dissolution on the ductility of SLMed AZ61 Mg alloy, the effect of solid solution heat treatment (T4) on the microstructure and mechanical properties of SLMed AZ61 was investigated and the kinetic model of β-Mg_17_Al_12_ dissolution of SLMed AZ61 magnesium alloy was established. According to the results, there is no significant change in the dissolution of the β-Mg_17_Al_12_ with an increase of temperature and time when the T4 temperature is lower than 410 °C. At the optimum solution heat treatment temperature of 410 °C, the dissolution rate is accelerated and the β-Mg_17_Al_12_ is completely dissolved after 2 h. In addition, the dissolution rate of β-Mg_17_Al_12_ decreases with the increase of dissolution time. The strength of SLMed AZ61 magnesium alloy decreases and the ductility increases as the T4 temperature increases. The strength of the specimens is reduced by grain coarsening (29.2 ± 3.7 μm), but the elongation is increased by 90% compared to SLMed AZ61, due to the effect of β-Mg_17_Al_12_ dissolution.

## 1. Introduction

During the last decade, magnesium alloys, particularly the AZ series (Mg-Al-Zn) magnesium alloy, have had a wide range of application prospects in the automotive, aerospace, and other industrial domains due to its high specific strength, high specific stiffness, excellent functional performance, and other advantages [1,2,3]. However, as a structural material, the large-scale application of magnesium alloy is still limited by its low strength and low plasticity [4].

In recent years, the selective laser melting (SLM) of magnesium alloys has attracted growing attention from and importance within industry, since as-built parts have higher strength and finer microstructure compared to cast counterparts. Compared with traditional manufacturing methods such as powder metallurgy and casting, SLM is more convenient in the production of complex structures [5], and the microstructure of the products is more uniform under the effect of rapid solidification due to the characteristics of layered manufacturing. The SLM-processed items have irreplaceable advantages in terms of high densities and dimensional accuracy [6,7,8,9,10]. Much research has been conducted on the application of the SLM process to various materials, such as stainless steels [11,12,13], nickel-based alloys [14,15], aluminum alloys [16,17,18], titanium alloys [19,20,21], magnesium alloys [22,23,24], WC/Co/Cr [25], etc. It is well-known that most of the studies have been devoted to the optimization of SLM process parameters [16]. However, the performance improvement of SLMed magnesium alloy by means of adjusting the process parameters is limited. The ultimate tensile and yield strengths of SLMed AZ91 magnesium alloy are reported to reach 296 MPa and 254 MPa, respectively, under the optimal process parameters. Still, the elongation is only 1.83%, which is much lower than that of the castings [26]. In the authors’ previous work, UTS and YS of SLMed AZ61 reached 287 MPa and 233 MPa, respectively, but the elongation was only 3.1%. Hence, the low elongation of SLMed magnesium alloy is an urgent problem that cannot be entirely solved by adjusting the process parameters.

It is well-known that post-heat treatments lead to microstructural evolution and a change in mechanical characteristics [27,28,29,30]. The SLM-prepared specimens have similar or higher hardness and tensile strength properties than conventionally fabricated specimens but with significantly lower elongation [26,31,32,33]. According to research by Guo et al. [34], after T4 heat treatment, the eutectic phase in AZ80 prepared by wire arc dissolved, the microstructure was more uniform, and the UTS and hardness values decreased, but the EL increased significantly. Wang et al. [35] showed that the η particles in an Al-Zn-Mg-Cu alloy prepared by SLM dissolved into the matrix after T6 heat treatment, and the mechanical properties were enhanced. Swetha et al. [36] showed that the heat treatment of cast AZ91 magnesium alloy should be at least 24 h, and that the heat treatment has a significant effect on the amount and distribution of the β-Mg_17_Al_12_. Additionally, the machinability of AZ91 Mg alloy can be improved by developing supersaturated grains and reducing the amount of β-Mg_17_Al_12_, which can be attributed to the solid solution strengthening. It has been reported that the elongation of SLM-prepared specimens can be improved by heat treatment [31,37]. However, based on the results of available studies, there is still a lack of research on the effect of heat treatment on the microstructure and mechanical properties, especially low elongation, of magnesium alloys fabricated by SLM.

It can be seen from our previous studies that HIP has a significant effect on improving the elongation of SLMed AZ61 magnesium alloy [33,37]. Different HIP temperatures result in varying amounts of β-Mg_17_Al_12_ dissolving into the matrix, as well as different improvements in ductility. However, the mechanism of dissolution of the β-Mg_17_Al_12_ in SLMed AZ61 magnesium alloys at high temperature is not clear. The impact of heat treatment on mechanical properties and microstructural evolution is the main topic of this work. Different temperatures and times of T4 heat treatments were applied to SLMed AZ61 magnesium alloy. The specimens created by SLM, HIP post-treatment, and T4 post-treatment were evaluated for microstructure, grain size, and mechanical characteristics. The relationship between the solution heat treatment and β-Mg_17_Al_12_ dissolution was clarified, and the influence mechanism of the solution heat treatment on ductility was explored. The dissolution kinetic model of β-Mg_17_Al_12_ was established and the β-Mg_17_Al_12_ dissolution characteristics were calculated theoretically, and the experimental basis for practical engineering applications was provided.

## 2. Material and Methods

### 2.1. Material and SLM Processing

Commercially atomized spherical Mg-6 wt%Al-1 wt% Zn (AZ61) alloy powders were used in this work. The particle size distribution ranged from 30 to 70 μm. The chemical composition of AZ61 magnesium alloy powder is shown in Table 1.

The experiments were carried out on an SLM machine FORWEDO LM-120 (Harbin, China). The laser of the SLM equipment was an IPG YLR-500 fiber laser. We used a zigzag scanning strategy in these experiments, so layer ‘n’ was scanned in the x direction and then layer ‘n + 1’ was rotated 90°. No preheating was used throughout the experiment. Table 2 shows the processing parameters used in the experiment. The size of the SLMed specimens was $10\times 10\times 10\;{\mathrm{mm}}^{3}$. More details regarding the SLM apparatus can be found in [33].

### 2.2. Solid Solution Heat Treatment

**Figure 1:**
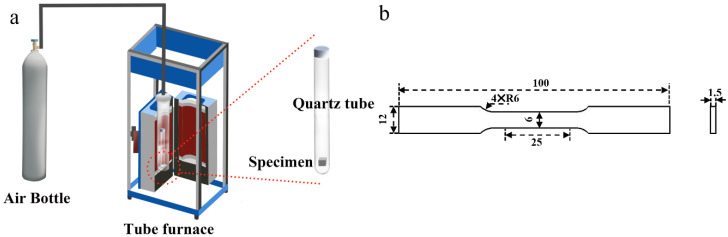
(**a**) Schematic of T4 heat treatment process; (**b**) Geometry of the tensile specimen.

The equipment used for heat treatment was a BLMT-1200 °C tube furnace. Before the experiment, the SLMed AZ61 magnesium alloy bulk and tensile specimens were first put into quartz tubes. The quartz tubes were filled with high-purity argon gas to avoid oxidation. The tube furnace was also filled with argon gas for atmosphere protection. The schematic of the T4 heat treatment process is shown in Figure 1a. The solid solution heat treatment test (T4) was then performed at 330 °C, 350 °C, 380 °C, and 410 °C. The heat treatment time was 2–10 h. Moreover, in order to investigate the dissolution characteristics of β-Mg_17_Al_12_ at shorter and longer times, heat treatment experiments at 410 °C for 5 min, 10 min, 15 min, 20 min, 30 min, 1 h, and 15 h were added; the experimental conditions of the heat treatment are shown in Table 3. The specimens were quenched in warm water immediately after heat treatment to retain the microstructure.

### 2.3. Microstructure and Mechanical Properties

A Netzsch differential scanning calorimeter (DSC) was used to test the thermal stability of the SLMed AZ61 magnesium alloy specimens. The heating speed was 10 k/min, and the cooling speed was 20 K/min. Specimens were ground, polished, and etched. The etching medium was 4% nitric acid alcohol, and the etching time was 5~15 s. The phase composition was determined by using X-ray diffraction (XRD; MXP21VAHF) with Cu Ka radiation. The microstructure of the specimens was characterized by SEM (JSM-6701F) and optical microscopy (Leica DM4M).

As shown in Figure 1b, tensile test specimens were first designed according to the ASTM B557M-10 standard. The surface of the specimens was ground and polished prior to tensile testing. For each fabrication condition, three tensile specimens were tested, using a CMT4105 material testing machine at a drawing velocity of 1 mm/min. The tensile properties were then determined according to the ASTM E-111 standards. Tensile tests were performed at room temperature with a strain rate of 1 × 10^−3^ s^−1^.

## 3. Results and Discussion

### 3.1. Microstructure

**Figure 2:**
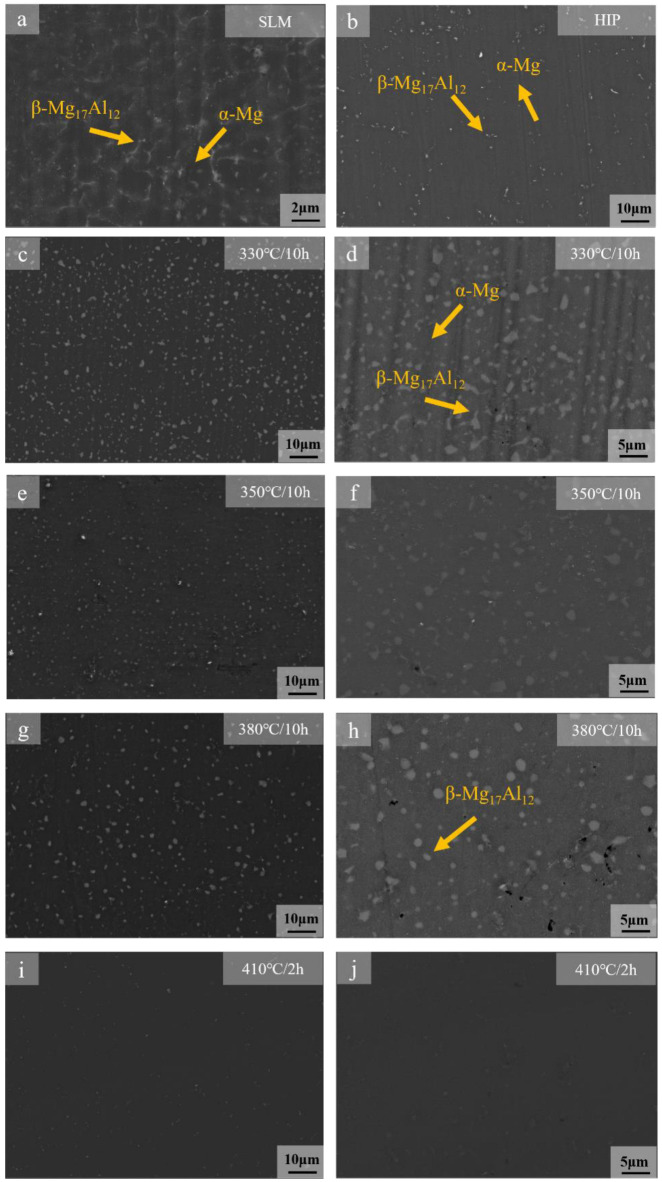
SEM images of the microstructure after different treatments: (**a**) SLMed AZ61; (**b**) HIP, 450 °C, 103 MPa, 3 h [37]; (**c**,**d**) 330 °C, 10 h; (**e**,**f**) 350 °C, 10 h; (**g**,**h**) 380 °C, 10 h; (**i**,**j**) 410 °C, 2 h.

The solid solution of the β-Mg_17_Al_12_ at different solution heat treatment temperatures and times is shown in Figure 2. The β-Mg_17_Al_12_ in SLMed AZ61 precipitated in a network form, while the β-Mg_17_Al_12_ dissolved into the matrix and forms diffuse fine particles after HIP treatment at 450 °C, 103 MPa, and 3 h. It is shown that the lattice-like Al-rich β-Mg_17_Al_12_ phase started to decompose at 330 °C and formed diffusely distributed bulk β-Mg_17_Al_12_. A similarly shaped β phase has been reported after annealing at 300 °C [38]. The β phase diffused in the α-Al matrix to form particles or spheroids, which is called the spheroidization transition, as shown in Figure 2c. The presence of balling particles in the microstructure can reduce the initiation of fatigue cracks. The area fraction of the β-Mg_17_Al_12_ had a slight decrease with increasing solid solution temperature when the temperature was increased from 330 °C to 380 °C. The area of the β-Mg_17_Al_12_ was calculated to be in the range of 4–6% by Image-Pro software. At 380 °C, a large amount of β-Mg_17_Al_12_ remained at 10 h. The β-Mg_17_Al_12_ phase dissolved completely within 2 h when the solid solution temperature was increased to 410 °C. Therefore, it can be assumed that the β-Mg_17_Al_12_ precipitated along the grain boundary at 410 °C for 2 h was dissolving faster and forming a single-phase supersaturated solid solution.

Image-Pro Plus 6.0 software was utilized to further analyze the size of β-Mg_17_Al_12_ particles under different conditions. First, for each sample, after grinding and polishing optical micrographs of three random selected positions were taken at a magnification of 2000×. Second, all the micrographs were transformed into grayscale, and a proper threshold value of grayscale was chosen using the segmentation function of the software to mark regions where the grayscale was the same as the β-Mg_17_Al_12_ phase. The diameter (mean) option was selected as the average size. Third, the software was utilized to set the scale of the images and the measurement range was limited to minimize the calculation error of the software. Finally, the size of the β-Mg_17_Al_12_ particles was determined by counting the diameter (mean) with the help of the statistics function. The calculation results show that 85 particles were calculated under SLM with an average size of 0.35 μm. The sizes of 176 particles were calculated at 330 °C/10 h, and the average size was 1.01 μm. The average sizes at 350 °C/10 h and 380 °C/10 h were 1.39 μm and 1.42 μm, respectively. Comparing with the β-Mg_17_Al_12_ in the as-cast alloys in literature [39], it can be seen that the size of the β-Mg_17_Al_12_ particles prepared by SLM were much smaller than those in the as-cast AZ61 alloy. This is due to the solubility of aluminum increasing with temperature; the maximum solubility of 12.6% can be reached at 437 °C [40]. The partial disappearance of the net β after 6 h of heat treatment is explained by the increase in the solubility limit at 410 °C. Many α-Mg grain boundaries were observed without β-Mg_17_Al_12_ with increasing heat treatment time. Therefore, heat treatment significantly changed the content and distribution of the β phase in SLMed AZ61 magnesium alloy.

**Figure 3:**
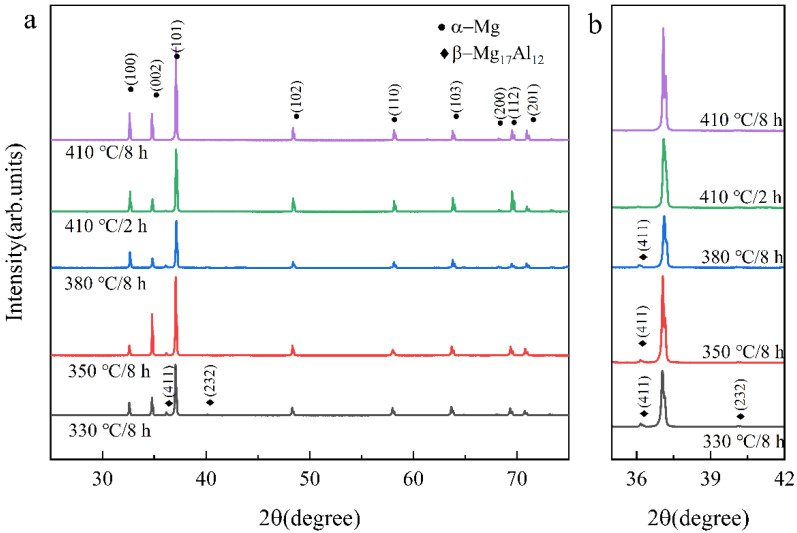
(**a**) XRD spectrum of SLM + T4 AZ61; (**b**) details in 35°–45°.

To further determine the phase composition at different temperatures and times, the phase compositions under 330 °C/8 h, 350 °C/8 h, 380 °C/8 h, 410 °C/2 h, and 410 °C/8 h were compared, as shown in Figure 3.

Figure 3a,b shows the SLM + T4 AZ61 magnesium alloy specimens consisting of two phases after solution heat treatment, α-Mg and β-Mg_17_Al_12_, at solution heat treatment temperatures below 410 °C. When the temperature was 330 °C, the β-Mg_17_Al_12_ (232) and (411) diffraction peak appeared in the XRD spectrum. The β-Mg_17_Al_12_ (232) diffraction peak disappeared as the temperature increased to 350 °C. However, the β-Mg_17_Al_12_ (411) diffraction peak still existed even as the solution heat treatment time reached 8 h at 380 °C. The β-Mg_17_Al_12_ (411) diffraction peak disappeared in 2 h when the temperature was raised to 410 °C. This indicates that a small amount of the β-Mg_17_Al_12_ began to dissolve at 350 °C, but it could not be completely dissolved, even if the heat treatment time was prolonged to 8 h; the β-Mg_17_Al_12_ was completely dissolved at 410 °C. The results of XRD are consistent with the microstructure in Figure 2. The reason for the dissolution of the β-Mg_17_Al_12_ at different temperatures was further studied in combination with the results of DSC experiments.

### 3.2. Thermal Stability of SLMed AZ61 Magnesium Alloy

**Figure 4:**
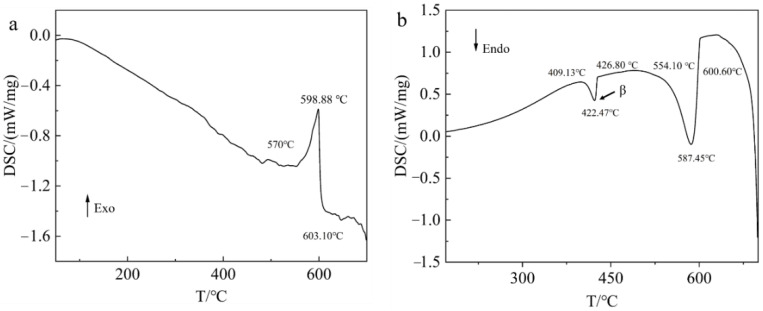
DSC curves of SLMed AZ61 magnesium alloy: (**a**) heating curve; (**b**) cooling curve.

In order to further analyze the theoretical dissolution temperature of the β phase, DSC tests were performed on SLMed AZ61 magnesium alloy specimens. The results are shown in Figure 4.

As shown in Figure 4a, the arrow represents the direction of exothermic process. The extrapolated initial melting temperature of α-Mg phase is 570 °C, and the extrapolated termination melting temperature is 603.10 °C. Their corresponding peaks, which represent the melting temperature of the α-Mg matrix, are at 598.88 °C. However, the β phase is not detected. This is because the specimen of SLMed AZ61 was used for the heating experiment. During the rapid solidification process of SLM, the amount of β phase precipitated is small, so the β phase starting melting temperature in the DSC temperature curve is not obvious, as shown in Figure 4a. Therefore, it is necessary to further analyze the evolution of the β phase in combination with the phase diagram and Figure 4b.

As shown in Figure 4b, the arrow represents the direction of endothermic process. According to the phase diagram [33], the solidification path of the AZ61 magnesium alloy at room temperature is:

L→L + α-Mg→α-Mg →α-Mg + β-Mg_17_Al_12_→α-Mg + β-Mg_17_Al_12_ + T

Combining with the phase diagram of AZ61 magnesium alloy [33], it can be seen that a small endothermic peak at 422.47 °C related to the transformation to β-Mg_17_Al_12_ phase is detected. The extrapolated initial crystallization temperature of the β-Mg_17_Al_12_ phase is 409.13 °C and the extrapolated termination crystallization temperature is 426.80 °C. The extrapolated initial crystallization temperature and the extrapolated termination crystallization temperature of the α-Mg matrix are 554.10 °C and 600.60 °C, respectively. Their corresponding peaks, which represent the melting temperature of the α-Mg matrix, are at 587.45 °C. In this temperature range, L→L + α-Mg occurs. Combining with the phase diagram of AZ61 magnesium alloy and Figure 4a,b, it can be seen that the transformation to the β-Mg_17_Al_12_ phase begins at 409.13 °C, reaches a maximum peak temperature at 422.47 °C, and ends at 426.80 °C. Therefore, the β-Mg_17_Al_12_ is completely dissolved at 410° C. When the solution heat treatment temperature (below 410 °C) is lower than the transformation temperature of the β-Mg_17_Al_12_ phase, the β-Mg_17_Al_12_ phase cannot be dissolved.

### 3.3. Evolution of Grain Size

**Figure 5:**
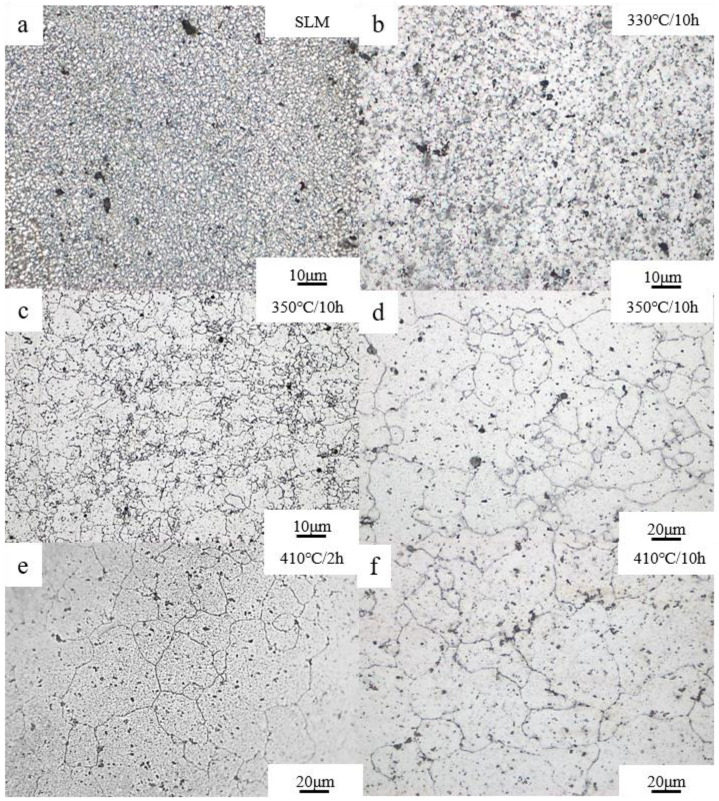
Optical microscopy microstructure of SLMed AZ61 prepared at different T4 temperatures and times: (**a**) SLMed AZ61; (**b**) 330 °C, 10 h; (**c**) 350 °C, 10 h; (**d**) high magnification of (**c**); (**e**) 410 °C, 2 h; (**f**) 410 °C, 10 h.

Determination of the optimal solution temperature and time is a concern of this study. The grain size measurements were conducted according to ASTM E112-13. Therefore, in order to ensure that the β-Mg_17_Al_12_ solid solution was complete and the grain size was minimum, we investigated the evolution of the microstructure and grain size when the solution temperature ranged from 330 °C to 410 °C and the solution time ranged from 2 h to 10 h. However, combined with the images of the microstructure, it can be seen that when the solution temperature is below 410 °C, the β-Mg_17_Al_12_ cannot be dissolved, even at 10 h, so it does not meet the optimal solution heat treatment conditions, and there are only images at 10 h under 330~380 °C. The effect of temperature on the grain size was investigated by comparing the grain size at 330~380 °C for 10 h. The evolution of grain size with solid solution temperature and time is shown in Figure 5. It can be seen that grain coarsening occurs with the increase in solution temperature. Referring to ASTM E112-13, grain size below level 12 was determined corresponding to the SLMed specimens and 330 °C/10 h, the average grain size was 1.9 ± 0.3 μm and 3.3 ± 1.3 μm, respectively. At 330 °C for 10 h, some grains grow, as shown in Figure 5b. As the solution temperature increased, the grains coarsened. At 350 °C/10 h, some of the grains’ size was level 8; the average grain size was 19.9 ± 1 μm. Some equiaxed grains formed at the grain boundaries and inside the grains after 350 °C for 10 h, indicating dynamic recrystallization of grains during the heat treatment at this temperature. Moreover, the grains did not grow with the prolongation of solid solution time at 410 °C. The grain size was level 5 at 410 °C/2 h and the average grain size was 29.2 ± 3.7 μm after 2 h and 29.4 ± 2.5 μm after 10 h. However, they were still much smaller than those of the as-cast specimen [41].

Therefore, combined with the results of microstructure and grain size, it can be seen that a large amount of β-Mg_17_Al_12_ solid solution dissolved into the α-Mg matrix at 410 °C; the role of the pinning effect on the inhibition of grain growth was reduced, resulting in an increase in grain size. Therefore, to ensure that the β-Mg_17_Al_12_ solid solution is complete and the grain size is minimum, it can be considered that 410 °C is the best solid solution temperature.

### 3.4. Mechanical Properties

**Figure 6:**
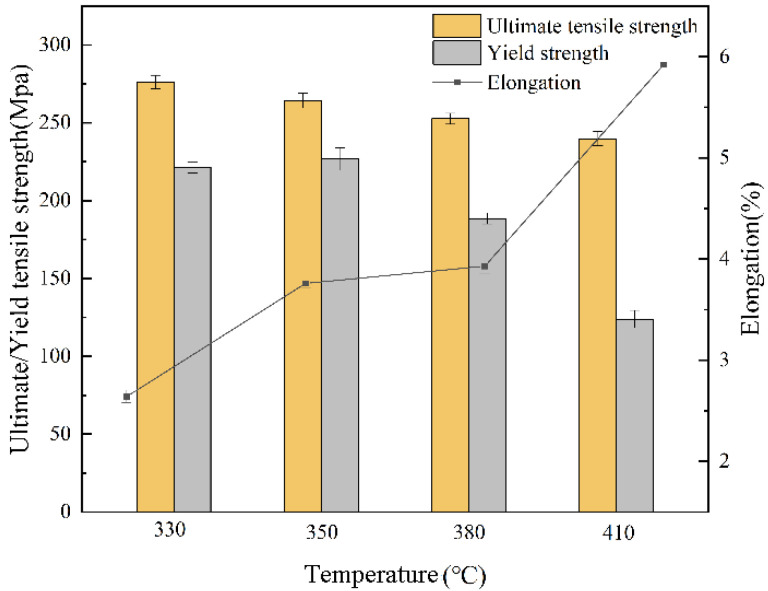
Comparison of mechanical properties of SLMed AZ61 magnesium alloy under different T4 treatment temperatures.

In order to investigate the effect of solution heat treatment on the mechanical properties of SLMed AZ61 magnesium alloy, the specimens after 10 h of heat treatment were selected for mechanical tensile experiments. Figure 6 shows the comparison of ultimate tensile strength (UTS), yield strength (YS), and elongation (EL) after 10 h of solid solution at four temperatures. As can be seen from Figure 5, the UTS decreased slightly with increasing solid solution temperature, from 276 ± 4 MPa (330 °C) to 240 ± 5 MPa (410 °C). The YS at 410 °C was reduced to 124 ± 6 MPa. However, the EL increased slightly from 2.6~3.9% (330 °C to 380 °C), and when the temperature increased to 410 °C, the EL increased significantly to 5.9%, which is 90% higher than SLMed AZ61 of 3.1% [37], but lower than the EL of 8.2% at 450 °C HIP [37].

The change in strength is mainly due to the combined effect of grain refining strengthening and solid solution strengthening, as shown in Equation (1) [42]

(1) $$\sigma_{\mathrm{sol}}=\sigma_{y0}+Z_{L}G{\left(\delta^{2}+\beta^{2}\eta^{2}\right)}^{2/3}c^{2/3}$$

where *σ_y_*_0_ is the yield stress of pure magnesium, *Z_L_* is a constant, *β* is between 1/20 and 1/16, *δ* is the size misfit parameter, *η* is the modulus misfit parameter, and *c* is the solute concentration. With the solid solution of the β-Mg_17_Al_12_ in the matrix leading to the increase in Al concentration, the solid solution strengthening effect is enhanced.

However, under the influence of high temperature, the grains become coarser and the grain boundary strengthening effect is weakened, leading to a decrease in strength, especially the decrease in yield strength, as shown in Equation (2) [42]

(2) $$\sigma =\sigma_{0}{\;+\;kd}^{\left(-1/2\right)}$$

where *σ* is the yield stress, *σ*_0_ is the friction stress when dislocations glide on the slip plane, *d* is the average grain size, and *k* is the stress concentration factor.

On the other hand, β-Mg_17_Al_12_ is only partially a solid solution at temperatures less than 410 °C, so the negative effect of β-Mg_17_Al_12_ on ductility is not eliminated completely, resulting in an increase in elongation, but still at a low level. It is reported that the ductility of the material can be enhanced due to the softening of the material under this kind of heat treatment, and the β-phase spheroidization eliminates the microstructural variations of the material, resulting in a more uniform microstructure [14,43]. However, β-Mg_17_Al_12_ is completely solidified into the matrix when the temperature is increased to 410 °C, and the source of cracks that are easily generated during the tensile process due to the mismatch between the β-Mg_17_Al_12_ and the α-Mg matrix is eliminated, and the ductility is improved, so the elongation is increased significantly. By comparing the mechanical properties of SLMed Mg alloy, solid solution heat-treated, and HIP-treated specimens, it has been confirmed that the dissolution of β-Mg_17_Al_12_ after solution heat treatment and HIP can effectively improve the ductility of SLMed Mg alloy, indicating that the precipitation of β-Mg_17_Al_12_ along the grain boundary will damage ductility during the tensile process due to the mismatch with α-Mg. In addition, closing the internal pores of the SLMed Mg alloy by HIP can further improve the ductility by increasing the specimen densities [44].

### 3.5. Fracture Behavior Analysis

**Figure 7:**
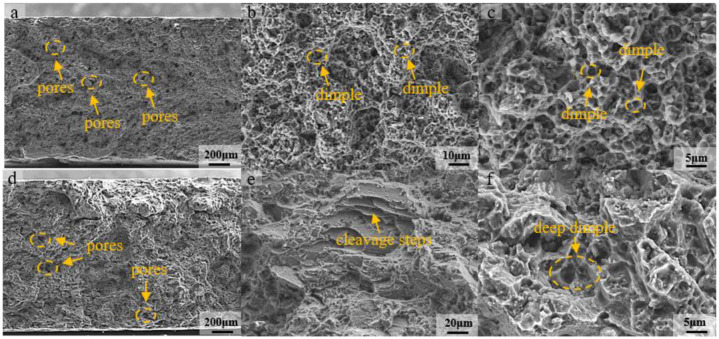
Fracture morphology at different T4 temperatures: (**a**–**c**) 330 °C; (**d**–**f**) 410 °C.

In order to further analyze the mechanical properties of the SLMed AZ61 magnesium alloy after solution heat treatment, the fracture morphology at two typical temperatures was selected for comparison. Therefore, the fracture morphology when the β-Mg_17_Al_12_ was not completely dissolved at 330 °C and 10 h and the fracture morphology when the β-Mg_17_Al_12_ was completely dissolved (410 °C, 10 h) were selected. The fracture morphology of the heat-treated specimens showed that some pores existed after heat treatment, as shown in Figure 7a,d. The fracture was mainly composed of small and shallow dimples at 330 °C, as shown in Figure 7b,c. This fracture morphology was similar to that of the SLMed AZ61 magnesium alloy, and the related results were published in a previous paper [33]. At this time, β-Mg_17_Al_12_ was not completely dissolved (Figure 2c) and the α-Mg grains were not fully grown (Figure 5c,d), so the strength and ductility of the specimen were similar to those of the SLMed AZ61 magnesium alloy. After 10 h at 410 °C, the β-Mg_17_Al_12_ in the specimen was almost completely dissolved, and the fracture morphology at 410 °C consisted of large and deep dimples with cleavage steps as shown in Figure 7e,f, indicating an increase in ductility and a mixed tough-brittle fracture mechanism, which is consistent with the experimental results. The β-Mg_17_Al_12_ of the SLMed part precipitated along the grain boundaries in a network, and the α-Mg and β-Mg_17_Al_12_ led to brittleness at the phase interface due to their different lattice structures. During the tensile process, the plastic deformation was mainly concentrated in the α-Mg matrix. After the solid solution heat treatment at 410 °C, the brittle-hard β-Mg_17_Al_12_ precipitated along the grain boundary was completely dissolved into the matrix, so the stress concentration at the interface between the α-Mg and β-Mg_17_Al_12_ was improved, and the crack source at the interface of α-Mg/β-Mg_17_Al_12_ was reduced; the ductility was thus improved [45,46]. The elongation is reported to be related to the pore size and densities of the specimens, so the elongation of the specimens after solution heat treatment was still lower than that of HIPed specimens [44].

### 3.6. Mechanism of the β-Mg_17_Al_12_ Dissolution

**Figure 8:**
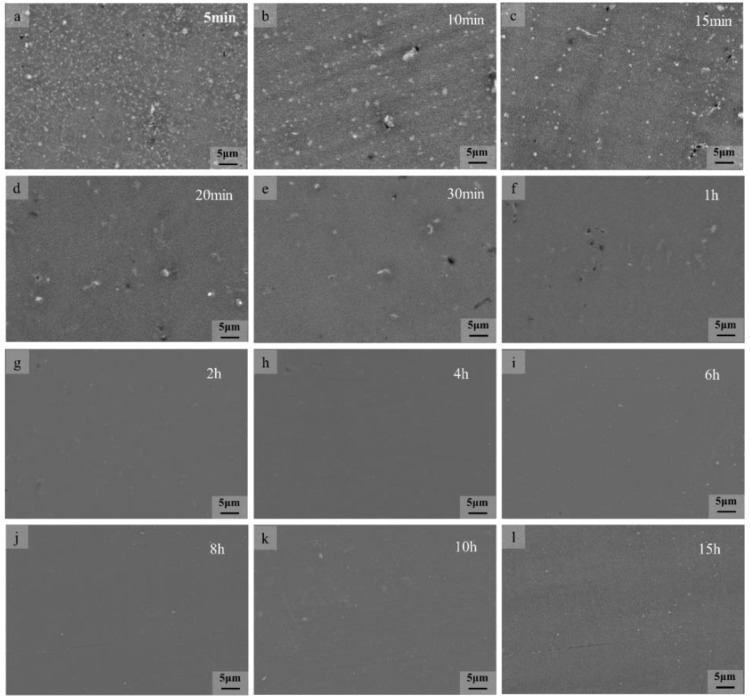
Dissolution of the β-Mg_17_Al_12_ at 410 °C for different times.

**Figure 9:**
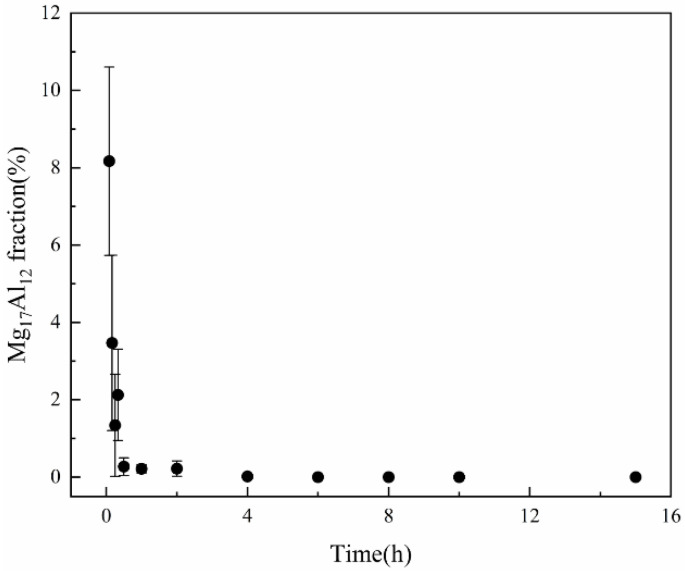
Variation of β-Mg_17_Al_12_ volume fraction with T4 time at 410 °C.

From the previous results, it is known that the β-Mg_17_Al_12_ in SLMed AZ61 magnesium alloy decomposes completely at 410 °C for 2 h. In order to further investigate the decomposition mechanism and the dissolution characteristics of the β-Mg_17_Al_12_ within 2 h, supplementary experiments were conducted, and the solid solution temperature was set at 410 °C for 5 min, 10 min, 15 min, 20 min, 30 min, 1 h, 2 h, 4 h, 6 h, 8 h, 10 h, and 15 h. The microstructure of the specimens was observed after the solid solution experiments, as shown in Figure 8. The variation of β-phase volume fraction with solid solution heat treatment time calculated by Image-Pro software is shown in Figure 9.

It can be seen from Figure 8 that at 410 °C, the dissolution of β-Mg_17_Al_12_ intensified with longer holding time and gradually solidified into the α-Mg matrix and the amount of the β-Mg_17_Al_12_ decreased. At 5 min, the microstructure consisted of equiaxed α-Mg grains and meshed β-Mg_17_Al_12_ with a small amount of diffuse granular β-Mg_17_Al_12_ precipitation (Figure 8a). Compared with the microstructure of the SLMed AZ61, a small part of the β-Mg_17_Al_12_ dissolved and part of the mesh disappeared after 5 min of solid solution heat treatment. At 10 min, the meshed β-Mg_17_Al_12_ was completely dissolved and the microstructure consisted of diffuse granular β-Mg_17_Al_12_ (Figure 8b); some of the β-Mg_17_Al_12_ particles grew slightly. After the solid solution heat treatment for 15 min, a large amount of granular β-Mg_17_Al_12_ solid solution dissolved into the α-Mg matrix. After the time was extended to 2 h, the β-Mg_17_Al_12_ was almost completely dissolved. As can be seen from Figure 9, the variation of β-Mg_17_Al_12_ volume fraction was consistent with the microstructure, which decreased rapidly in the first 30 min and decreased from 8.1% to 1.2% when the time was from 5 min to 30 min. The β-Mg_17_Al_12_ was completely dissolved after 2 h. Considering the grain size, β dissolution, and economic efficiency, 410 °C and 2 h can be considered the optimal solid solution temperature and time.

### 3.7. Kinetic Model of Dissolution

It can be seen that β-Mg_17_Al_12_ undergoes a phase transition during the solution heat treatment at 410 °C and its dissolution rate varies at different times. Therefore, it is necessary to investigate the dissolution mechanism of the β phase, so theoretical calculations of β-phase dissolution kinetics are performed using the JMAK equation [47], as shown in Equation (3).

(3) $$f=1-\exp\left(-kt^{n}\right)$$

where *f* is the fraction dissolved at time *t* at constant temperature, *k* is the temperature-dependent isothermal rate constant, and *n* is the time exponent. The values of *k* and *n* can be calculated by ln(ln [1/(1 − *f*)]) and ln(*t*), and Equation (2) can be obtained by transforming Equation (4),

(4) $$\ln\ln\frac{1}{1-f(t)}=\ln k+n\ln t$$

**Figure 10:**
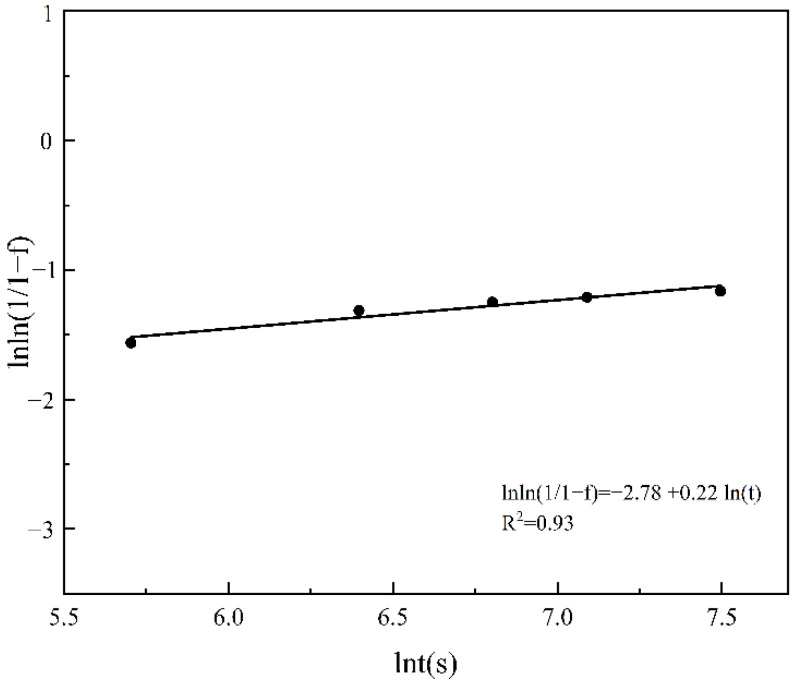
Plot of ln{ln[1/(1 − *f*)]} versus ln(*t*).

As the dissolution of the β phase is a complex process, the kinetic analysis of β-phase dissolution is simplified according to the dissolution characteristics of the β phase. From Figure 8 and Figure 9, it can be seen that β-Mg_17_Al_12_ dissolves faster in the first 1 h, and the change is no longer significant after 1 h. Therefore, combining the XRD results (Figure 3) and SEM images (Figure 2), it can be seen that β-Mg_17_Al_12_ dissolves most significantly within 2 h. Thus, the experimental interval within 1 h was shortened to 5~10 min intervals, and the times were 5 min, 10 min, 15 min, 30 min, and 1 h. Considering that the most significant stage of dissolution can better show the mechanism of β-Mg_17_Al_12_ dissolution, the first five sets of experimental data were selected for plotting. We hoped to reduce the error introduced by the data from the non-dissolution stage. Therefore, the β-Mg_17_Al_12_ dissolution for the first 1 h was plotted by ln{ln[1/(1 − *f*)]} against ln(*t*) to obtain the values of *n* and *k*, as shown in Figure 10.

Thus, the kinetic model of β-Mg_17_Al_12_ dissolution of AZ61 prepared by SLM is established. The fitted curve is ln{ln[1/(1 − *f*)]} = −2.78 + 0.22ln(*t*),R^2^ = 0.93. In addition, the value of R^2^(0.93) is slightly lower than R^2^ = 0.94 in the literature on the β-Mg_17_Al_12_ dissolution mechanism of AZ80 magnesium alloy [47]. Our purpose in presenting this part of the data is mainly to clarify the trend of β-Mg_17_Al_12_ dissolution. Combining the results of the fitted curve, we can see that *k* and *n* are $6.2\times 10^{2}{\;s}^{-1}$ and 0.22, respectively, where *n* is between 0 and 1, indicating that the dissolution rate of the β phase decreases with the increase in dissolution time. This is related to the distribution of Al in the α-Mg matrix near the precipitated phase of β-Mg_17_Al_12_. According to the Mg-Al phase diagram, the equilibrium concentration of Al in the α-Mg matrix is 11% at 410 °C. During the dissolution of β-Mg_17_Al_12_, the concentration of Al in the matrix near the β-Mg_17_Al_12_ increases. Therefore, in order for β-Mg_17_Al_12_ to dissolve more, Al must diffuse from the region near the β phase to the surrounding α-Mg matrix in order to accommodate more Al. However, this process is slow because the diffusivity of Al in magnesium is small (between 10^−13^ m^2^/s and 10^−14^ m^2^/s) [48], leading to a decrease in the dissolution rate of β-Mg_17_Al_12_ precipitates with increasing dissolution time.

## 4. Outlook

Based on the previous research on the effect of hot isostatic pressing on SLMed AZ61 magnesium alloy, the effect of high temperatures on the dissolution of the β-Mg_17_Al_12_ and the change of elongation after the dissolution of the β-Mg_17_Al_12_ were further explored in order to solve the problem of high strength and low elongation of SLMed magnesium alloy.

The main focus of this research was on the dissolution of the β-Mg_17_Al_12_ at different temperatures and the changes in the strength and elongation values with the dissolution of the β-Mg_17_Al_12_. However, it was important to establish the relationship between different temperatures, strain rates, and strength, and more mechanical properties at different temperatures and strain rates need to be studied. By analyzing the mechanical properties under various strain rates and temperatures, the temperature-stress constitutive model also needs to be established, which ultimately ensures the objectivity and reliability of the model.

In addition, through literature investigation, we found that magnesium alloys can exhibit plastic instability, i.e., sawtooth flow or the Portevin–Le Chatelier (PLC) effect under plastic deformation conditions, which presents a sawtooth wave on the stress–strain curve. The PLC effect is related to the strain rate, temperature, and grain size. Rodriguze et.al. [49] revealed that the finer the grain size of the alloy, the easier the PLC effect occurs. Geiselman et al. [50] showed that sawtooth flow occurs only when magnesium alloys are subjected to solid solution heat treatment. However, other scholars believed that magnesium alloys can exhibit the PLC effect at both high temperature and room temperature [51]. Feng et al. indicated that the tensile curve of AZ31B magnesium alloy exhibits an obvious PLC effect when the strain rate is higher than 1397 s^−1^, and the strength and elongation are significantly higher than those under the condition of low strain rate. So far, there are few reports on the PLC effect of magnesium alloys under high strain rate conditions, and the related mechanism is still unclear. Especially for the fine grains of magnesium alloys prepared by SLM, the research on the PLC effect at different strain rates and temperatures is still lacking. Therefore, the stress–strain curves and related mechanisms of SLMed magnesium alloys at different strain rates and temperatures need to be further studied.

## 5. Conclusions

In order to further investigate the dissolution characteristics of β-Mg_17_Al_12_ at high temperature and to elucidate the mechanism of the effect of solution heat treatment on β-Mg_17_Al_12_ in SLMed AZ61 magnesium alloy, solution heat treatment experiments have been carried out. Heat treatment for the as-fabricated AZ61 magnesium at 330 °C, 350 °C, 380 °C, and 410 °C at 2~10 h has been investigated. In addition, supplementary experiments were conducted for 5 min, 10 min, 15 min, 20 min, 30 min, 1 h, and 15 h at 410 °C. The grain size, microstructure evolution, and mechanical properties of the specimens were examined, and the dissolution kinetic theoretical calculations of the dissolution properties of the β-Mg_17_Al_12_ were performed.

(1) The dissolution of the β-Mg_17_Al_12_ and grain sizes were related to the solid solution temperature and time. At temperatures below 410 °C, β-Mg_17_Al_12_ did not dissolve significantly with changes in solid solution temperature and time (even if 10 h), and the decomposition of the net-like β-Mg_17_Al_12_ precipitated along the grain boundaries formed a bulk structure from 330 °C. The almost complete dissolution of β-Mg_17_Al_12_ occurred after just 2 h at 410 °C and the dissolution rate was significantly higher at 410 °C.
(2) The grain size increased from 3.3 ± 1.3 μm to 29.2 ± 3.7 μm at 330~410 °C. The grain size was without significant growth (29.4 ± 2.5 μm) after 10 h, but was still much smaller than that of the as-cast specimen.
(3) The kinetic model of β-Mg_17_Al_12_ dissolution of AZ61 prepared by SLM was established, and the dissolution characteristics of β-Mg_17_Al_12_ at 410 °C were analyzed theoretically. The dissolution rate of β-Mg_17_Al_12_ decreased with the increase of dissolution time. Moreover, the optimal solid solution heat treatment temperature and time were 410 °C and 2 h.
(4) The mechanical properties study showed that the strength of SLMed AZ61 magnesium alloy decreased and the ductility increased as the solution heat treatment temperature increased. Additionally, β-Mg_17_Al_12_ dissolution was beneficial to improving elongation. At 410 °C, the UTS was 240 ± 5 MPa and the YS decreased to 124 ± 6 MPa under the combined effect of solid solution strengthening and grain coarsening. However, the EL increased to 5.9%, which was due to the reduction of crack generation caused by the mismatch between the β-Mg_17_Al_12_ and the α-Mg matrix after the dissolution of the β-Mg_17_Al_12_, and the EL increased by 90% compared to SLMed AZ61 specimens. In addition, HIP can further close the internal pores, leading to greater elongation.

## Figures and Tables

**Table 1:** The chemical composition of AZ61.

Elements	Mg	Al	Zn	Mn	Si	Fe	Cu	Ni
Wt.%	balance	6.25	1.24	0.27	0.06	0.03	0.01	0.01

**Table 2:** SLM processing parameters.

Processing Parameters	Value
Laser power, P	150 W
Scanning speed, v	400 mm/s
Laser beam spot size, D	70 mm
Hatch spacing, H	60 mm
Powder layer thickness, T	40 mm

**Table 3:** Experimental conditions of heat treatment.

Methods	SLM	HIP	T4 Heat Treatment			
T/°C	-	450	330	350	380	410
P/MPa	-	103	-	-	-	-
t/h	-	3	2~10	2~10	2~10	2~10 h 5 min, 10 min, 15 min, 20 min, 30 min, 1 h, 15 h

## Data Availability

All the data are available within the manuscript.
